# The volatilome – investigation of volatile organic metabolites (VOM) as potential tumor markers in patients with head and neck squamous cell carcinoma (HNSCC)

**DOI:** 10.1186/s40463-018-0288-5

**Published:** 2018-07-03

**Authors:** Philipp Opitz, Olf Herbarth

**Affiliations:** 1 Institute of Hygiene, Hospital Hygiene and Environmental Medicine, University Hospital Leipzig, Johannisallee 34, 04103 Leipzig, Germany; 2 Institute of Environmental Medicine and Hygiene, Faculty of Medicine, University of Leipzig, Liebigstr. 27, 04103 Leipzig, Germany

**Keywords:** Tumor marker, Urinary metabolites, Volatile organic metabolites (VOM), Non-invasive diagnostics, Head and neck squamous cell carcinoma (HNSCC), Solid-phase micro extraction (SPME)

## Abstract

**Background:**

Different organisms such as bacteria, molds and humans produce and release a relative unknown class of metabolites which are responsible for the individual olfactory pattern. These volatile organic metabolites (VOM) represent a kind of biosignature that reflects the sum of all multifactorial influences, including genetics, environmental factors, nutritional and disease status. As a result of pathological processes the individual body odor can be influenced by newly produced or altered compositions of the VOM. Until now, human VOM have been detected in various body media, such as on the skin, in the exhaled air as well as in body fluids such as saliva, mother’s milk, sweat, blood and urine.

**Methods:**

In this retrospective case-control study urinary VOM of 53 therapy-naive patients with head and neck squamous cell carcinoma (HNSCC) and 82 healthy controls were semi-quantified by headspace solid-phase micro extraction (SPME) gas chromatography (GC) mass spectrometry (MS). At first, the procedure was optimized in respect to the extraction parameters. By using *Student’s t*-test significant differences in the VOM pattern with the corresponding *p*-values were obtained. For multivariate metabolite pattern recognition the hierarchical cluster analysis by *Ward* was applied, followed by the supervised partial least squares-discriminant analysis (PLS-DA).

**Results:**

In total 81 VOMs could identified in the urine samples of all study participants, of which 25 were significantly increased and of which were 10 significantly reduced in HNSCC-patients compared to the controls. In addition, the multivariate statistics confirmed that on the basis of the renal excreted pattern of the volatile metabolites a high discrimination can be carried out between patients with a tumor in the head and neck region and controls. The substance group of the saturated, unbranched aldehydes makes a substantial contribution in this context.

**Conclusions:**

The systematic pattern analysis of urinary VOMs appears to have potential clinical application as a diagnostic tool for cancer, especially HNSCC.

**Electronic supplementary material:**

The online version of this article (10.1186/s40463-018-0288-5) contains supplementary material, which is available to authorized users.

## Background

According to the World Health Organization (WHO) volatile organic compounds (VOC) are outlined as carbonaceous substances with a boiling point range between 50 and 260 °C. These compounds result from natural but also from anthropogenic origin [38] and they are ubiquitous, which means that they are found both indoors and outdoors. However, the pollution indoors is given a special importance [39]. The entry sources are versatile: besides building materials, furnishings, floor coverings, paints and lacquers, as well as activities such as smoking, cooking and hobbies play a decisive role [15, 24].

In addition to pollutants from the indoor air, hundreds to thousands of VOC are produced by the human organism and released to the environment. On the one hand, they are responsible for the typically individual body odor and on the other hand they reflect the metabolic conditions of the respective individual. Therefore, a further term has been established: “volatile organic metabolites” (VOM) [34, 35]. These volatile metabolites can be detected in various media of the body, such as on the skin and in the exhaled air, as well as in body fluids such as saliva, mother’s milk, sweat, blood and urine [1, 18]. VOM represent a kind of biosignature which depicts the sum of the multifactorial influences to an individual, e.g. genetics, environmental factors, nutritional status and disease status [2]. As a result of pathological processes the individual body odor can be influenced by newly produced or altered compositions of the VOM, including infections or endogenously caused metabolic disorders [14]. In this case, the VOMs are not bound to be formed exclusively endogenously in the organism, they can also occur from exogenous sources and thus they reflect the exposome. They can be derived from food, from inhaled pollutants from indoor air and from stimulants such as tobacco smoke and alcohol consumption. In addition, growing microorganisms produce a series of volatile secondary metabolites, so-called microbial volatile organic compounds (MVOC), which are again metabolized by the colonized host or interact with the metabolites of the host and generate therefrom new VOCs. This leads, for example, to a foul smell of exhaled air in respiratory infections [6].

For this reason, it is of great interest to derive disease-specific VOMs for the medical diagnosis of different diseases, which appear as olfactory biomarkers. Conclusions about certain VOMs can be drawn by understanding the pathophysiological mechanisms, which provide insights for appropriate therapeutic treatment approaches. These markers are formed by diseased or healthy cells in the organism in response to the disease [37]. In reference to tumor diseases a change in the microbial colonization of the organism is assumed, which modulates the pattern of the VOMs. A number of studies have shown that the VOMs can be used to discriminate between different diseases [5, 23, 36]. In recent years, cancer-specific VOMs are increasingly being discussed their role as tumor markers [11, 22, 26, 27, 32].

Considering that head and neck cancers represent the sixth most common cancer worldwide with more than 550.000 cases and 380.000 deaths per year the search towards appropriate tumor markers for an early and reliable diagnostics must be expedited vehemently. The risk factors for development of head and neck squamous cell carcinomas (HNSCC) include smoking, excessive alcohol consumption, and infection with human papillomavirus or Epstein-Barr virus. Amongst other things, high expectations are being placed on the elucidation of the human metabolome, since the metabolites pass through all complex enzymatic pathways and thus are excreted as end products of the metabolism.

## Methods

### Patients and study population / collection of urine samples

For the extraction of the VOMs from the urine material a retrospective case-control study including total *n* = 53 patients with pathohistological confirmed HNSCC and *n* = 82 healthy volunteers served as a basis. Patients were recruited at the head and neck (ENT) clinic of the University Hospital Leipzig between 03/2009 and 09/2011; details see Table 1. The localization of the primary tumors comprised larynx, hypopharynx, oropharynx, and oral cavity. In all cases the urine sample was obtained before starting the curative therapy. Healthy controls were matched according to the criteria depict age, sex and the predominant lifestyle factors in HNSCC, i.e. tobacco smoking and alcohol consumption. It has to be pointed out that the matching regarding life style factors was not possible in detail for each case. The participants of the control group have been obtained from different studies: LIFE (Leipziger Forschungszentrum für Zivilisationserkrankungen – Leipzig research center for diseases of civilization), recruitment at the ENT-Clinic (patients without cancer, inflammatory diseases or pregnancy), and an internal study for creation of reference values. All urine samples were taken after obtaining the patients written informed consents as approved by the local institutional review board (votes of the ethics committee of the Medical Faculty of the University Leipzig (No. 201–10-12,072,010 & No. 202–10-12,072,010)).

**Table 1:** Clinical characteristics of the HNSCC group (UICC staging system = Union internationale centre le cancer)

Mean age	61.0	
Sex	n	(%)
Male	43	(81.1%)
Female	10	(18.9%)
UICC stage		
I	4	(7.5%)
II	6	(11.3%)
III	10	(18.9%)
IVA	30	(56.6%)
IVB	3	(5.7%)
IVC	–	(0%)
Localisation		
Oropharynx	28	(52.8%)
Hypopharynx	10	(18.9%)
Larynx	10	(18.9%)
Oral cavity	5	(9.4%)
Tobacco consumption		
Yes	42	(79.2%)
No	11	(20.8%)
Alcohol consumption		
Yes	78	(77.4%)
No	14	(22.6%)

The urines were filtered immediately using a 0.2 μm membrane cellulose acetate filter (Sartorius) to remove any sediment potentially containing epithelial cell, erythrocytes, bacteria and proteins which disturb the further analysis. The filtered urine samples were stored at minus 20 °C no longer than 8 weeks until processed.

### Reagents

Helium N50 and nitrogen N50 (both purity 99.999%) were purchased from AirLiquid (Düsseldorf, Germany). Deionized water was acquired from a Milli O plus purification system (Merck Millipore, Bedford, MA, USA). 99.99% Suprapur sodium chloride (NaCl) and 25% pure hydrochloric acid (HCl) were received from Merck (Darmstadt, Germany) respectively AppliChem (Darmstadt, Germany).

### Headspace solid-phase micro extraction (SPME)

The extraction of the VOM was done by the fast and solvent-free method of solid-phase microextraction (SPME). This method involves the sampling, extraction and concentration of the analytes in a single step and was first described by C. Arthur and J. Pawliszyn [3]. The extraction can be carried out either directly from the urine sample or in the headspace. In this case, the enrichment in the headspace was preferred, since this handling is both the more gentle application for the SPME fiber and minimizes the aqueous residues reaching for the gas chromatograph (GC) column. A Carboxen / PDMS coated fiber from Supelco (Bellefonte, PA, USA) was applied for the adsorption of the VOM. Therefore, the following sentences are appropriate: 'At first, the procedure was optimized in respect to the extraction parameters. The optimization parameters can be found in the Additional file 1.'

### Sample preparation

The frozen urine samples were completely thawed at room temperature and gently homogenized. On the basis of the above-described results, 10 ml of each urine sample were transferred into a 20 ml headspace vials from Labsolute (Th. Geyer, Renningen, Germany) and 2 g of NaCl were added, resulting in a concentration of 0.2 g/ml. With continuous stirring with a PTFE-coated 11 mm magnetic stirrer (VWR International, Darmstadt, Germany), the NaCl was completely dissolved and then the pH was adjusted to pH 2 using 25% HCl. The HCl was added using a 10 μl microliter syringe (Hamilton, Bonaduz, Swiss) while simultaneously monitoring the pH by means of the pH meter sension1 (HachLange, Berlin, Germany). Subsequently, the headspace vials were sealed with the magnetic screw caps (Th. Geyer, Renningen, Germany) and placed in the fully automatic PAL-xt sampler (Chromtech, Idstein, Germany).

### Separation of urinary VOM

The VOM were extracted among continuous stirring for 30 min at 50 °C. The enriched components were desorbed from the SPME fiber in the SSL injector for 6 min at 250 °C. The application of the sample to the GC column was applied in splitless mode. Between each separate analysis, the SPME fiber was purified and conditioned at 290 °C for 10 min in a stream of nitrogen. The desorbed analytes from the SPME fiber were first separated by a GC 7890a on a fused silica DB-SELECT 624UI capillary column (60 m × 0.25 mm × 1.4 μm) and then detected by means of a mass spectrometric detector 5975c (column and GC/MS from Agilent, Santa Clara, CA, USA). Helium was used as the carrier gas at a constant gas flow of 2 ml/min. The oven program was as following: 0.3 min at 30 °C; then rise at 10 °C/min to 100 °C; after rise at 3 °C/min to 130 °C; then rise at 5 °C/min to 240 °C; hold for 10.2 min at final temperature.

### Evaluation of the chromatograms

Due to the extent of detected VOMs from the urine samples as well as to the partially unknown components and non-available standard substances a special calibration with the corresponding quantification was omitted. By the help of the ChemStation software version F.01.01.2317 (Agilent, Santa Clara, CA, USA) the evaluation were carried out semiquantitatively by the integrated peak areas. The separated peaks from the chromatograms of the 135 investigated subjects were incorporate into a separate database by means of their retention times and the specific mass-to-charge ratio (m/z) from the ion spectrum via the individual target and qualification ions. Only analytes were included in the further investigations, identified by the NIST 08 spectra library with at least 80% accuracy.

### Statistics

The statistical analysis and representation of the results has been done using Microsoft Office Excel® 2010 (Microsoft Deutschland GmbH, Unterschleißheim, Germany), SPSS 20 (IBM Corporation, Armonk, NY, USA), and STATISTICA 10 (StatSoft Inc. 2011, Tulsa, OK, USA).

For standardization, the peak areas of the identified substances were related to the respective mean value of the individual variables. To examine the distribution and significant differences the *Kolmogorow-Smirnow-*test (*KS*-test) and the *Student’s t*-test (*t*-test) were used. For the recognition of patterns in the sophisticated data structure, the hierarchical cluster analysis according to the *Ward*-method was applied. In this context as the distance measure the *Euclidean* distance was used.

In addition, the multivariate procedure of the partial least squares-discriminant analysis (PLS-DA) was applied to the data set of the VOM based on the nonlinear iterative partial least squares (NIPALS) algorithm.

For all statistical calculations, a significance level of α < 0.05 was established.

## Results

A total of 306 differentiated peaks from the chromatograms of the 135 measured urine samples were recorded with the use of the ChemStation® software. After differentiating with the peaks assigned to the blank measurements approximately one-third of the original 306 detected peaks disappeared. As a further criterion, an equal to or greater 80% hit probability by the National Institute of Standards and Technology (NIST) 08 spectra library were determined for the identification of an unknown metabolite. Furthermore, the respective metabolite both in the control group and in HNSCC-patients should not be below a frequency of at least 90%. This finally resulted in a database of 81 VOM, which are summarized in the following Table 2.

**Table 2:** List of the identified VOM using the NIST 08 spectra library

	Name of the volatile organic metabolite (VOM)	CAS-number	Chemical formula	t_R_ [mins]	m/z	frequency [%]	
						HNSCC	Healthy
1	Furan	110–00-9	C_4_H_4_O	5.53	68	100	100
2	Propanal	123–38-6	C_3_H_6_O	5.65	58	100	100
3	Acetone	67–64-1	C_3_H_6_O	5.90	43	100	100
4	3-Pentanol	584–02-1	C_5_H_12_O	5.97	59	100	92.7
5	1-Propanol	71–23-8	C_3_H_8_O	7.21	31	98.1	93.9
6	2-Methylfuran	534–22-5	C_5_H_6_O	7.46	82	100	100
7	3-Methylfuran	930–27-8	C_5_H_6_O	7.74	82	100	100
8	2-Butanone	78–93-3	C_4_H_8_O	7.84	43	100	100
9	2-Methylbut-3-en-2-ol	115–18-4	C_5_H_10_O	8.24	71	100	100
10	Acetic acid	64–19-7	C_2_H_4_O_2_	8.69	43	100	100
11	Benzene	71–43-2	C_6_H_6_	9.00	78	100	100
12	3-Methylbutanal	590–86-3	C_5_H_10_O	9.16	44	98.1	100
13	Thiophene	110–02-1	C_4_H_4_S	9.20	84	98.1	97.6
14	2-Methylbutanal	96–17-3	C_5_H_10_O	9.37	41	100	100
15	2-Ethylfuran	3208–16-0	C_6_H_8_O	9.64	81	100	100
16	2,5-Dimethylfuran	625–86-5	C_6_H_8_O	9.84	96	100	100
17	2-Pentanone	107–87-9	C_5_H_10_O	10.04	43	100	100
18	2,4-Dimethylfuran	3710–43-8	C_6_H_8_O	10.15	96	98.1	100
19	Methyl methacrylate	80–62-6	C_5_H_8_O_2_	10.23	55	100	100
20	2-Ethenylfuran	1487–18-9	C_6_H_6_O	10.65	105	100	100
21	Tetrahydro-2,2,5,5-tetramethylfuran	15,045–43-9	C_8_H_16_O	11.48	43	100	100
22	Dimethyl disulfide	624–92-0	C_2_H_6_S_2_	11.56	94	100	100
23	3-Methyl-2-pentanone	565–61-7	C_6_H_12_O	11.99	43	100	100
24	Toluene	108–88-3	C_7_H_8_	12.01	91	100	100
25	2-Methylthiophene	554–14-3	C_5_H_6_S	12.23	97	100	98.8
26	2-Ethyl-5-methylfuran	1703–52-2	C_7_H_10_O	12.53	95	100	100
27	3-Hexanone	589–38-8	C_6_H_12_O	12.93	43	100	100
28	2,3,5-Trimethylfuran	10,504–04-8	C_7_H_10_O	13.12	43	100	100
29	2-Hexanone	591–78-6	C_6_H_12_O	13.20	43	100	100
30	Hexanal	66–25-1	C_6_H_12_O	13.47	44	100	100
31	m-Cresol	108–39-4	C_7_H_8_O	14.01	108	100	100
32	2-Acetyl-5-methylfuran	1193–79-9	C_7_H_8_O_2_	14.31	109	100	100
33	5-Methyl-3-hexanone	623–56-3	C_7_H_14_O	14.71	57	100	100
34	4-Methyl-3-hexanone	17,042–16-9	C_7_H_14_O	14.94	57	100	100
35	Ethylbenzene	100–41-4	C_8_H_10_	15.38	91	100	100
36	2-Hexenal	6728–26-3	C_6_H_10_O	15.90	41	100	100
37	4-Heptanone	123–19-3	C_7_H_14_O	16.19	43	100	100
38	2-Methylbutanoic acid	116–53-0	C_5_H_10_O_2_	16.41	74	100	100
39	3-Heptanone	106–35-4	C_7_H_14_O	14.74	57	100	100
40	Styrene	100–42-5	C_8_H_8_	16.85	104	100	100
41	2-Heptanone	110–43-0	C_7_H_14_O	17.05	43	100	100
42	Heptanal	111–71-7	C_7_H_14_O	17.43	70	100	98.8
43	4-Methyl-2-heptanone	6137–06-0	C_8_H_16_O	19.00	43	100	100
44	3-Methyl-2-heptanone	2371–19-9	C_8_H_16_O	19.25	43	100	100
45	2-Ethenyltetrahydro-2,6,6-trimethyl-(2H)-pyran	7392–19-0	C_10_H_18_O	19.45	139	100	100
46	2-Methyl-5-(methylthio)-furan	13,678–59-6	C_6_H_8_OS	20.19	128	100	100
47	Dimethyl trisulfide	3658–80-8	C_2_H_6_S_3_	20.61	126	100	100
48	Benzaldehyde	100–52-7	C_7_H_6_O	20.95	77	100	100
49	Terpinolen	586–62-9	C_10_H_16_	21.35	93	98.1	100
50	1,4-Cineole	470–67-7	C_10_H_18_O	21.42	43	100	100
51	Octanal	124–13-0	C_8_H_16_O	21.61	43	100	100
52	D-Limonene	5989–27-5	C_10_H_16_	21.79	68	98.1	100
53	o-Cymol	527–84-4	C_10_H_14_	21.93	119	100	100
54	1,3,5-Trimethylbenzene	108–67-8	C_9_H_12_	22.21	105	100	100
55	Dihydro-5-methyl-2(3H)-furanone	108–29-2	C_5_H_8_O_2_	22.29	56	98.1	98.8
56	1,8-Cineole	470–82-6	C_10_H_18_O	22.34	43	100	100
57	Tetrahydro-2,2-dimethyl-5-(1-methyl-1-propenyl)furan	7416–35-5	C_10_H_18_O	22.60	43	100	100
58	Phenol	108–95-2	C_6_H_6_O	23.42	94	100	100
59	2,6-Dimethyl-7-octen-2-ol	18,479–58-8	C_10_H_20_O	24.13	59	100	100
60	Benzyl alcohol	100–51-6	C_7_H_8_O	24.50	108	100	100
61	Dehydro-p-cymol	1195–32-0	C_10_H_12_	24.63	117	100	100
62	Tetrahydrolinalool	78–69-3	C_10_H_22_O	25.06	73	92.5	96.3
63	Linalool	78–70-6	C_10_H_18_O	25.30	71	100	100
64	Nonanal	124–19-6	C_9_H_18_O	25.45	57	100	100
65	3,4-Dimethyl-2,5-furandione	766–39-2	C_6_H_6_O_3_	25.70	39	100	100
66	4-Tolualdehyde	104–87-0	C_8_H_8_O	25.81	91	100	100
67	p-Cresol	106–44-5	C_7_H_8_O	26.63	107	100	100
68	± − 4-Acetyl-1-methylcyclohexene	70,286–20-3	C_9_H_14_O	27.13	43	100	100
69	1-(1,4-dimethyl-3-cyclohexen-1-yl)-ethanone	43,219–68-7	C_10_H_16_O	27.74	109	98.1	100
70	Camphor	76–22-2	C_10_H_16_O	28.20	95	100	97.6
71	4-Terpineol	562–74-3	C_10_H_18_O	28.43	71	98.1	100
72	α-Terpineol	98–55-5	C_10_H_18_O	29.07	59	100	100
73	1,3-Di-tert-butyl-benzene	1014–60-4	C_14_H_22_	29.80	175	100	100
74	DL-Carvone	99–49-0	C_10_H_14_O	31.16	82	100	100
75	4-tert-Butyl-2-chlorophenol	98–28-2	C_10_H_13_ClO	33.49	169	100	100
76	4-tert-Butylphenol	98–54-4	C_10_H_14_O	33.57	135	100	100
77	1,2-Dihydro-1,1,6-trimethylnaphthalene	30,364–38-6	C_13_H_16_	33.82	157	100	100
78	β-Damascenone	23,726–93-4	C_13_H_18_O	35.03	69	100	100
79	7,8-Dihydro-α-ionone	31,499–72-6	C_13_H_22_O	36.00	136	96.2	100
80	3,4-Dehydro-β-ionone	1203–08-3	C_13_H_18_O	36.77	43	100	100
81	α-Calacorene	21,391–99-1	C_15_H_20_	38.86	157	100	100

**Fig. 1:**
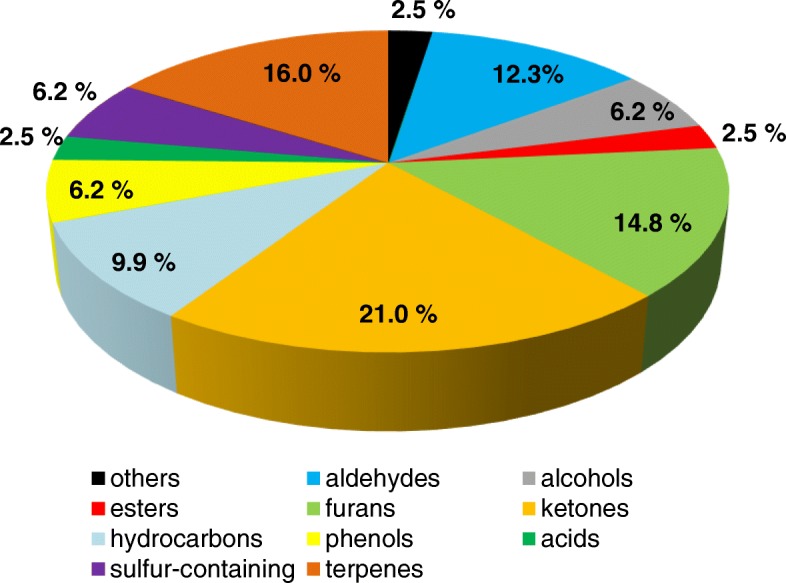
Relative distribution of the VOMs according to the chemical classes

The VOMs were classified according to their corresponding chemical classes and the relative distributions, illustrated in Fig. 1. The 81 identified VOMs included the 10 groups of aldehydes, alcohols, esters, furans, ketones, hydrocarbons, phenols, acids, sulfur-containing compounds, and terpenes. For a better overview, a separate classification into the aliphatic and aromatic hydrocarbons was omitted. The largest portion of the class of substances excreted in urine was ketones (21.0%), followed by terpenes (16.0%) and furans (14.8%). On the other hand, the esters and acids each of 2.5% make the smallest contribution of the chemical compounds in the urine. In addition, it must be stated that an undoubted classification of individual compounds is not to be carried out consistently without restriction. For example, some terpenes, such as α-terpineol and linalool occurring as monocyclic respectively acyclic monoterpene alcohols, belong by their functional group also to the substance class of alcohols. The phenols, which have been listed as an independent group, can formally also be classified to the alcohols.

### Differences between controls and HNSCC-patients

**Fig. 2:**
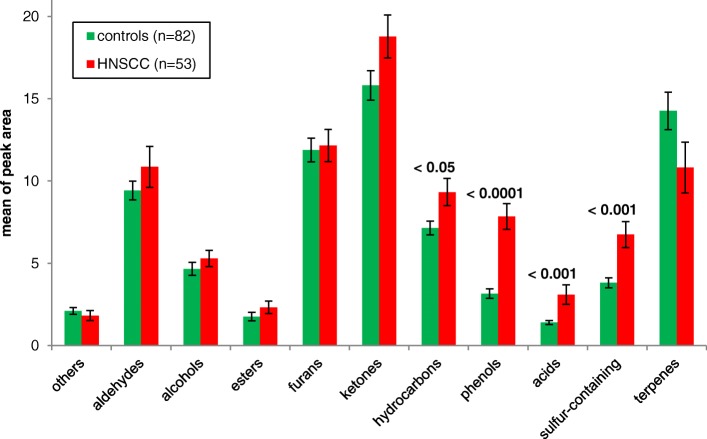
Differences in the metabolite patterns relative to the chemical classes in the urine of HNSCC-patients (*n* = 53) and controls (*n* = 82)

Considering the renal pattern of the VOMs, summarized in the corresponding chemical classifications of patients with a tumor disease in the head and neck region and of healthy controls, there are deviations in the concentration which are characterized by the peak areas of the analyzed compounds (Fig. 2).

In the urine of the patients, the classes of hydrocarbons, phenols, acids and sulfur-containing compounds are significantly increased compared to healthy controls. In addition, the aldehydes and ketones are enhanced in the urine of patients, even if this relationship has no significant character. At least a trend for the ketones with *p* = 0.053 can be observed. On the other hand, an increased excretion of terpenes in the urine could be detected in the control group, although this relationship is not significant.

**Fig. 3:**
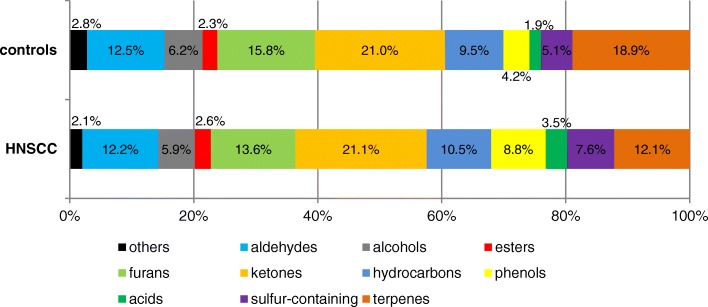
Relative excretion pattern of the VOM according to the both groups

In addition, for a simplified visual comparison of the pattern of the emitted compounds in the urine the individual classes of chemicals were compared with the total sum of all occurring substance classes and the HNSCC-patients were plotted against the healthy study population. At becomes apparent that the increase in the proportion of furans and terpenes in the urine of the healthies against the patients, while the spectrum in patients is shifted to the phenols and sulfur-containing compounds (Fig. 3).

**Fig. 4:**
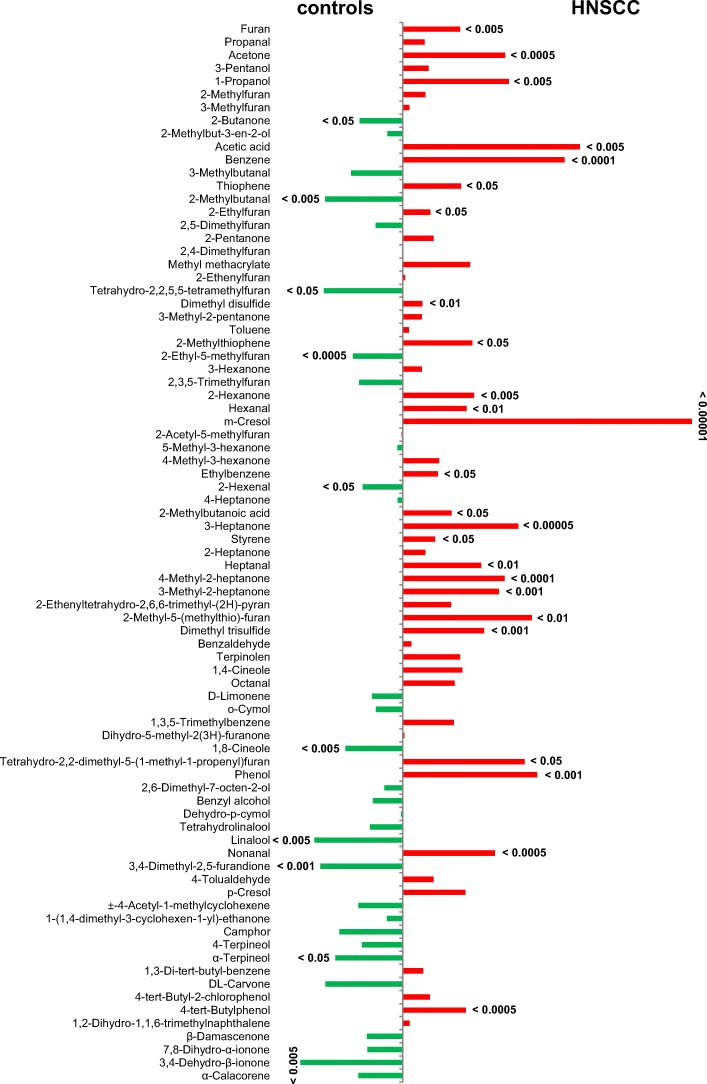
Significant differences between controls (left – green) and HNSCC patients (right – red) in the VOM pattern

For the derivation of specific metabolite markers, which may indicate a tumor disease in the urine, the difference of the means between the patient and control group were plotted against each other. The green bars on the left side symbolize a higher difference in mean values ​​in the control group, whereas the red bars represent a higher mean in the group of patients (Fig. 4). In addition, the significant differences between the two groups were distinguished based on the comparison of the mean values. The 25 VOMs m-cresol, 3-heptanone, benzene, 4-methyl-2-heptanone, acetone, 1-propanol, nonanal, 4-tert-butylphenol, phenol, 3-methyl-2-heptanone, dimethyltrisulfide, 2-hexanone, ethanoic acid, furan, hexanal, 2-methyl-5-(methylthio) furan, heptanal, dimethyldisulfide, 2-methylthiophene, tetrahydro-2,2-dimethyl-5-(1-methyl-1-propenyl)furan, 2-methylbutyric acid, styrene, 2-ethylfuran, ethylbenzene and thiophene were significantly increased in the urine of patients with malignant neoplasia.

In contrast, it can be noted that 10 VOMs were significantly reduced in the urine of HNSCC-patients compared to the healthy controls. These components included 2-ethyl-5-methylfuran, 3,4-dimethyl-2,5-furanedione, 3,4-dehydro-β-ionone, 2-methylbutanal, linalool, 1,8-cineol, 2-butanone, α-terpineol, tetrahydro-2,2,5,5-tetramethylfuran and 2-hexenal.

### Multivariate analyzes of VOM

**Fig. 5:**
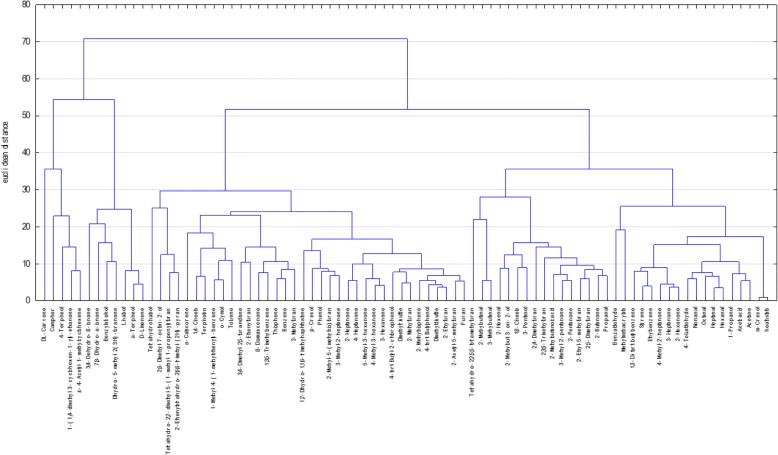
Dendrogram of the 81 analyzed VOMs and the target variable “health state” (right side)

First, the standardized peak areas of the 81 captured VOMs from the urine of the study cohort as well as the target variable were incorporated for the systematic analysis. Target variable was declared as a “health state” in the dendrogram. In order to integrate all existing cases into the investigation, missing values ​​in the data set were replaced by the corresponding mean value of the variable. Figure 5 shows the dendrogram of the cluster analysis performed, with the location of the target variable at the far right of the graph. As appears from the figure, m-cresol has obviously the smallest distance measure towards the target variable and forms with it the smallest cluster at the very edge on the right side of the diagram. In the next step, the target variable “health state” is combined with a series of variables to the next larger cluster. In fact, this cluster is composed of two further clusters, whereby the variables acetone, ethanoic acid, 1-propanol, hexanal, heptanal, octanal, nonanal, 4-tolualdehyde are contained on the right side. In the left cluster, the variables 2-hexanone, 3-heptanone, 4-methyl-2-heptanone, ethylbenzene, styrene and 1,3-di-tert-butylbenzene are combined into a common group. The terpenes DL-carvone, camphor and 4-terpineol are located farthest from the target variable, which are found in the dendrogram shown at the extreme left margin.

Furthermore, the hierarchical cluster analysis shows that separate VOMs belonging to the same chemical class are combined to the corresponding clusters. For example, the group of aldehydes around hexanal, heptanal and octanal forms their own cluster. This is reflected in further chemical compounds, such as the alcohols, ketones and terpenes.

**Fig. 6:**
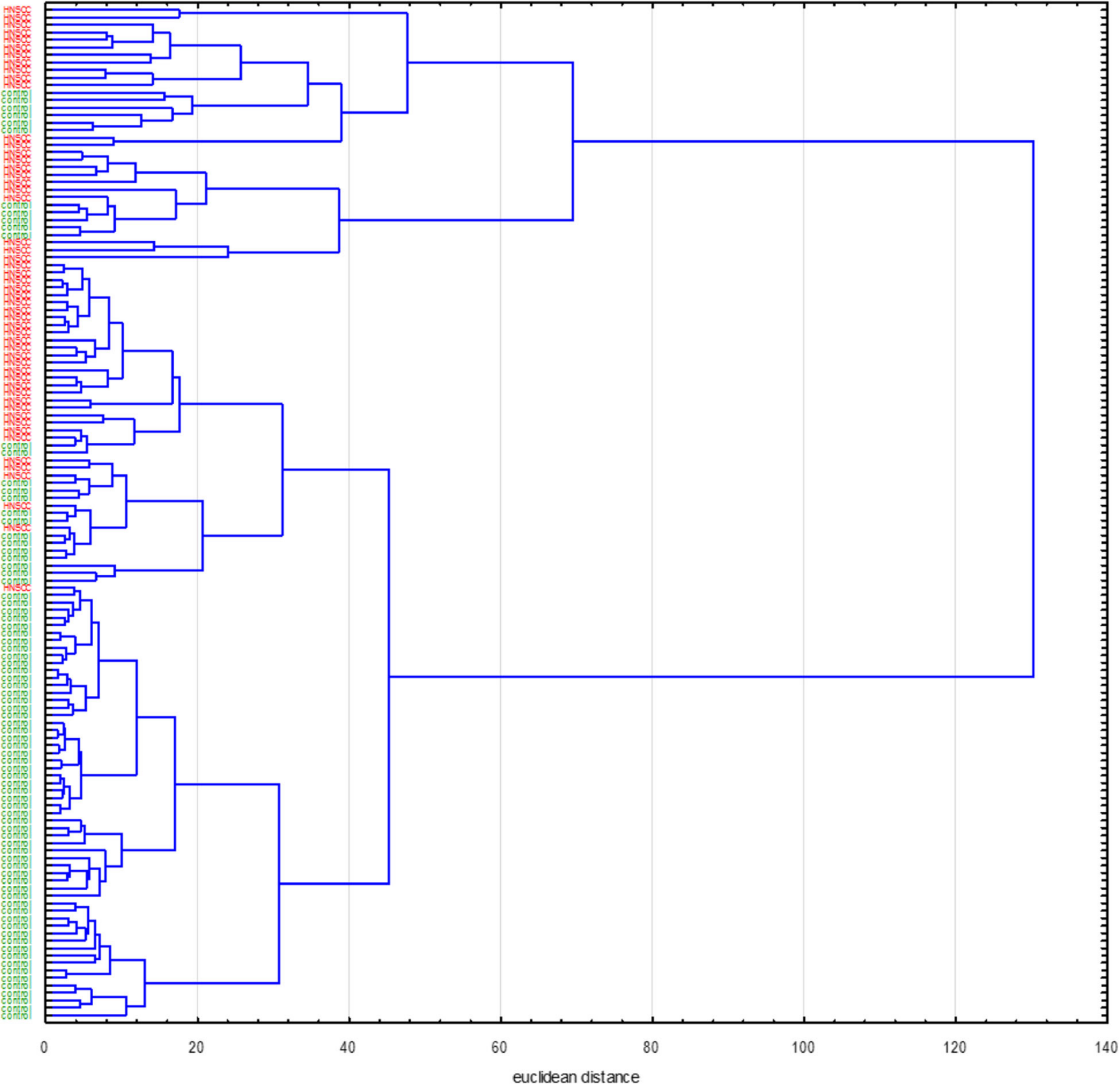
Dendrogram of all 135 cases (HNSCC - red, controls - green)

In the further course the 135 cases of the entire study population were subjected to the cluster analysis. In this evaluation only the statistically significant VOMs from the univariate analysis were included as variables, which are reproduced in Fig. 4. Analog to the previous cluster analysis, missing values in the data matrix were replaced by the corresponding mean values to allow that all 135 study participants were involved. The results of the subordinate hierarchical cluster analysis are shown in Fig. 6. For a simplified overview, the HNSCC-patients at the left end were marked red and the controls were marked green. From the dendrogram it can be seen that the controls and patients with a tumor disease are not generally divided into two superordinate clusters. There are areas in the middle of the tree structure where healthy people and HNSCC-patients are grouped as a cluster based on similarity structures in the pattern of the incoming variables. Despite this, there are wide areas within the dendrogram where the patients can be grouped into individual clusters due to their similarities in the VOM pattern. This effect is also characteristic for the controls, especially at the lower end of the tree structure in Fig. 6.

As another multivariate procedure for discriminating between HNSCC-patients and controls, as well as the derivation of potential tumor markers which are responsible for the separation between the two case groups, the supervised PLS-DA was applied which considered the assignment of the two groups. The state of health of the 135 study participants (0 = controls, 1 = HNSCC) served as a categorical, dependent variable, whereas the significant VOM from the univariate analysis were entered as steady predictors into the model. To ensure the quality of the created model a 7-fold cross-validation was carried out.

**Fig. 7:**
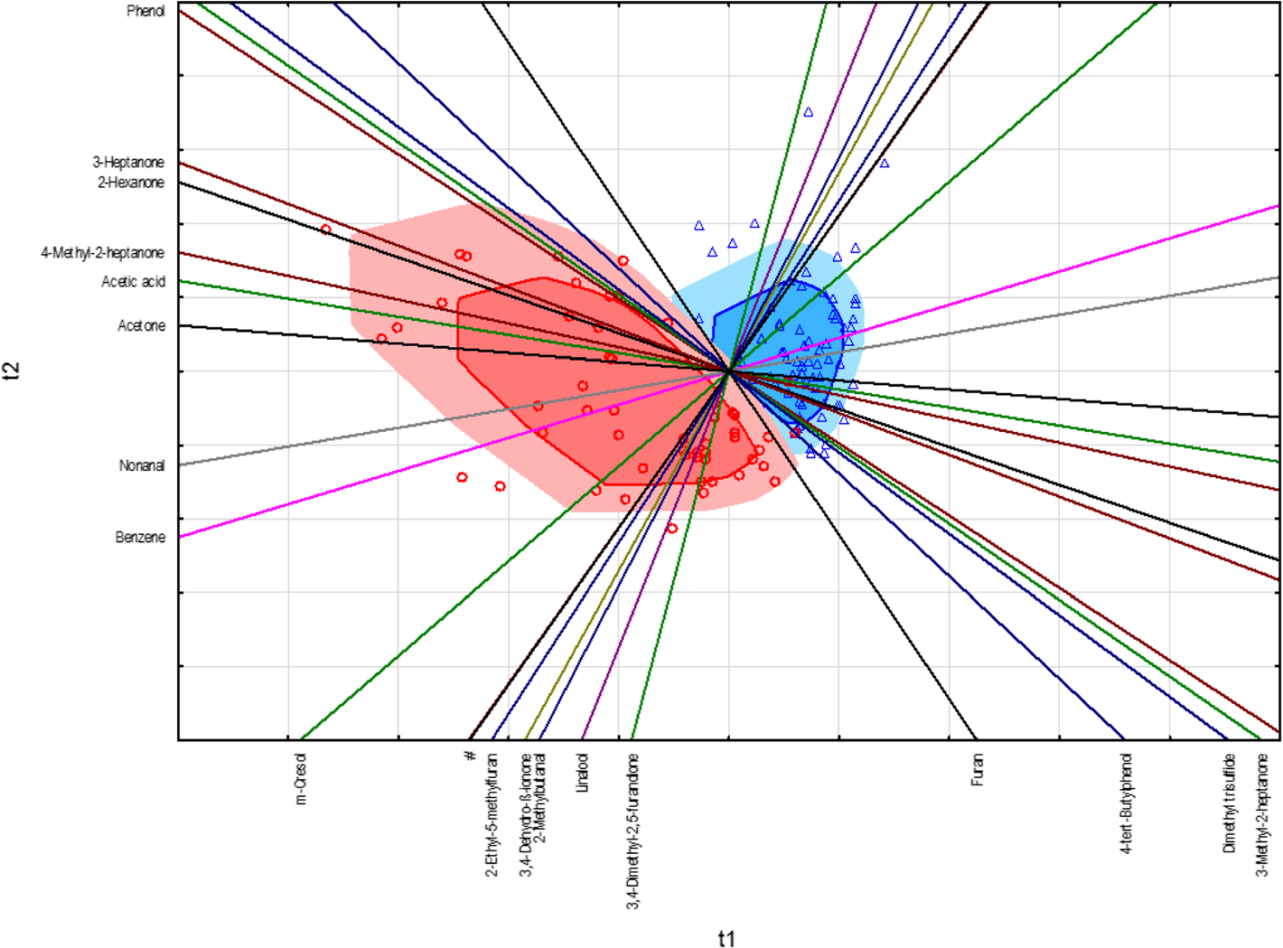
Standardized biplot of the PLS-DA between HNSCC-patients (red circles) and controls (blue triangles), (# - 1,8-cineole and 1-propanol)

The results of the PLS-DA are illustrated in a standardized biplot (Fig. 7). This allows presenting the cases sorted by groups together with the incoming variables in a two-dimensional projection. The patients with a tumor in the head and neck region are shown as red circles in the study cohort, while the controls are characterized by blue triangles. On the other hand, the variables are described by the course of the colored axes in the standardized biplot, from which are derived the charges of the individual variables from the respective component axis t1 or t2. For example, acetone has a high charge towards component t1 since the variable axis is almost parallel to the corresponding component axis t1. In addition, the correlation relations of the variables among each other can be determined from the variable axes. For example, a very strong correlation between 1,8-cineol and 1-propanol can be found in the biplot, which was summarized by the very close neighboring position in the legend. After a closer look at the two variables in the weight plot, which is not listed separately, a negative correlation relation is shown. The standardized biplot demonstrates that the discrimination is generally possible, taking into account the VOM pattern between the two groups, even if two of the tumor patients were wrongly classified into the control group. In addition, the HNSCC-patients show a greater spread, while the controls are more homogeneous in their pattern. For the classification between the two groups, m-cresol, benzene, nonanal and acetone seem to be the most likely VOM to identify patients with malignant neoplasia in the head and neck region.

## Discussion

The importance of the human VOM is being increasingly discussed to what extent these are able to reflect the current metabolic situation and accordingly the health condition [1, 2, 8, 34]. So far, the focus has been laid on the detection of exhaled volatile metabolites by means of respiratory gas analyzes for the diagnosis of very different disease pictures, e.g. allergic asthma [9], chronic obstructive pulmonary disease (COPD) [6], renal dysfunction [17], liver cirrhosis [10], hepatocellular carcinoma [30], mama carcinoma [19, 27], and bronchial carcinomas [11, 28, 41]. Above all, the exhaled biomarker 2-methyl-1,3-butadiene, better known as isoprene, represents a promising target of clinical diagnostics. Isoprene is the basic unit of terpenes and is generated during the cholesterol biosynthesis in the mevalonate pathway [20]. Furthermore, the identification of specific VOM signatures is also encourage in the stool as well as other body fluids, such as, blood, sweat secretion, saliva and urine. Specifically, urine is particularly suitable for the detection of prognostic biomarkers since a large number of metabolic end products are excreted in the urine which passes through the complexity of all biochemical pathways in the human organism. Besides, using urine as sample matrix is a non-invasive procedure, thereby avoiding a superfluous physical burden or unexpected side effects due to the examination for the patient.

The results of this study demonstrated that patients with squamous cell carcinoma in the head and neck region exhibit characteristic patterns of VOMs in the urine compared to healthy subject groups. Above all, the volatile metabolites belonging to the chemical compounds of the aldehydes, ketones, hydrocarbons, phenols, acids and sulfur-containing compounds have been found increased in the urine of HNSCC-patients. On the other hand, the substance class of the terpenes showed an inverse behavior. Concretely, for the compounds m-cresol, 3-heptanone, benzene, 4-methyl-2-heptanone, acetone, 1-propanol, nonanal, 4-tert-butylphenol, phenol, 3-methyl-2-heptanone, dimethyl trisulfide, 2-methyl-5- (methylthio) furan, heptanal, dimethyl disulfide, 2-methylthiophene, tetrahydro-2, 2-dimethyl-5-(1-methyl-1-propenyl) furan, 2-methyl-butyric acid, styrene, 2-ethylfuran, ethylbenzene and thiophene were determined significantly higher levels in the urine of the patient population. While the following VOMs presented significantly reduced; 2-ethyl-5-methylfuran, 3,4-dimethyl-2, 5-furanedione, 3,4-dehydro-β-ionone, 2-methylbutanal, linalool, 1,8-cineol, 2-butanone, α-terpineol, tetrahydro-2,2,5,5-tetramethylfuran and 2-hexenal. Furthermore, the multivariate statistics confirmed that on the basis of the renal excreted pattern of the volatile metabolites a classification between the two investigated study cohorts can be carried out. In addition to m-cresol and benzene, the substance group of the saturated, unbranched aldehydes shows a significant contribution. This substance class formed also a defined cluster separated from the other chemical compounds in the dendrogram due to their similarity in the renal excretion pattern (Fig. 5). In addition to the hydrocarbons ethane and pentane, which are frequently used for respiratory gas-mediated tumor diagnostics [8, 14], the aldehydes are also used as volatile biomarkers for the lipid peroxidation of unsaturated fatty acids [13, 31]. Their detection is associated with the occurrence of oxidative stress which is initiated as a result of a disturbed equilibrium between free oxygen radicals and antioxidants. This is due to the overproduction of reactive oxygen species (ROS), such as the superoxide, hydrogen peroxide and the hydroxyl radical, which are produced intracellularly as byproducts of the cellular respiration in mitochondria and secreted into the cytoplasm of the cell [33]. In addition to the generation of oxidative stress, which is responsible for a number of pathological processes, including diabetes, inflammatory and neurodegenerative diseases, early aging and the initiation of carcinogenesis [29], ROS also play a central role as secondary messengers in regulatory processes within the cell, as well migration, proliferation and programmed cell death. Numerous studies have proved the intracellularly increased concentrations of ROS in tumor cell lines as well as in the microenvironment of tumors, where they promote the further tumor progression [7, 21]. Furthermore, ROS are capable of causing the oxidation of biologically central molecules such as DNA, RNA, and proteins. Besides, they initiate the lipid peroxidation of polyunsaturated omega-3 and omega-6 fatty acids, which serve as basic components of cell membrane lipids, whereas among others unsaturated and saturated aldehydes are formed as secondary degradation products [12, 25]. In this context malondialdehyde, 4-hydroxynonenal, propanal and hexanal have been described as suitable markers [4, 16]. Up to now an increased release of aldehydes were found in the exhaled air [19], blood [40] and urine [12] of patients with various tumor diseases.

On the one hand, from a scientific point of view, on the other hand, to advance an early clinical diagnosis, there is a marked interest in assigning the individual VOMs to their respective place of origin to derive any characteristic tumor markers or biomarkers which reflect an unfavorable exposure. Up to now, the development processes of the VOM have not been known very well. In general a distinction is made between endogenous and exogenous volatile metabolites. Exogenous results from ingested food, from inhaled air pollutants and microbial colonization. A following unpublished publication will following to examine the origins of the humans VOM with exogenous influences, such as alcohol consumption and tobacco smoke.

## Additional file

Additional file 1: **Figure S1** Effect of the extraction time on the peak area sum of all analytes at 50 °C with a CAR / PDMS fiber. **Figure S2** Correlation between the extraction and addition of NaCl, as well as the shift of the pH value. (DOCX 335 kb)

## Abbreviations

CI: Confidence interval
COPD: Chronic obstructive pulmonary disease
GC: Gas chromatography head and neck (ENT) clinic
HNSCC: Head and neck squamous cell carcinoma
MS: Mass spectrometry
MVOC: Microbial volatile organic compounds
NIPALS: Nonlinear iterative partial least squares
NIST: National Institute of Standards and Technology
NN: Neuronal network
OR: Odds ratio
PCA: Principal component analysis
PLS-DA: Partial least squares - discriminant analysis
ROC: Receiver operation characteristic
ROS: Reactive oxygen species
SPME: Solid-phase micro extraction
VOC: Volatile organic compounds
VOM: Volatile organic metabolites
