# Supplementary material for: The volatilome – investigation of volatile organic metabolites (VOM) as potential tumor markers in patients with head and neck squamous cell carcinoma (HNSCC)
Source: J Otolaryngol Head Neck Surg. 2018 Jul 3;47:42. doi: 10.1186/s40463-018-0288-5 (PMC6029417; doi:10.1186/s40463-018-0288-5)
Supplement: Supplementary file 1 — Figure S1 Effect of the extraction time on the peak area sum of all analytes at 50 °C with a CAR / PDMS fiber. Figure S2 Correlation between the extraction and addition of NaCl, as well as the shift of the pH value. (DOCX 335 kb) [file 40463_2018_288_MOESM1_ESM.docx]

## Additional file

*Optimization of headspace SPME conditions*

The principle is based on the equilibrium distribution of the analytes between the liquid and gaseous phases, as well as between the gaseous phase and the used SPME fiber. After setting equilibrium between urine sample and gas space, as well as gas space and extraction fiber, the concentration of the analytes to be extracted in the fiber is proportional to its concentration in the liquid sample matrix. The adjustment of the equilibrium distribution is essentially influenced by the temperature and time of the extraction. Figure 1 shows the relationship between different extraction times and the enriched amount of the analytes, which was characterized as the sum of all analyzed peak areas in the chromatogram, at a fixed temperature of 50°C. Up to an extraction time of 30 minutes, a clear increase in the total peak areas can be observed, whereas longer extraction times show no improvement. In addition, a reduction in the peak area sum could be observed for longer extraction times, possibly due to the back-diffusion effects of individual components. Therefore, an extraction time of 30 minutes at an extraction temperature of 50°C was selected, since higher temperatures lead to a degradation of the sample matrix.

Besides the extraction time and temperature, the use of a magnetic stirrer, the addition of salt and the adjustment of the pH value also affect the adjustment of the equilibrium. The addition of NaCl leads to an over-saturation of the liquid sample matrix, which reduces the solubility, in particular for non-polar components, in the aqueous phase and thus leads to an enrichment in the upside headspace.

On the other hand, significantly more components were enriched from the gas phase above the urine matrix during the shift of the pH value, both in the alkaline and acidic range. Particularly the shift to the acidic environment increases the efficiency of the extraction procedure. By adjusting the pH of the aqueous solution, the distribution coefficients for the dissociable components were changed. The SPME fiber extracted only the undissociated mutation of an acid or base in the contained solution. Furthermore, the addition of HCl to the urine samples initiates an acidic hydrolysis of the excreted glucuronic acid and sulfate conjugates from the phase II reaction of the biotransformation. Thus, more potential analytes were available in their unconjugated form for the transition to the gas phase. The relationship between the different sample handlings and the number of extracted substances, as well as their concentrations, expressed by the sum of all the peak areas analyzed in the total ion concentration (TIC) of the chromatogram, is demonstrated in Figure 2. The potentiation of the extraction after the addition of 0.2 g/ml NaCl and the adjustment of the pH value to the acidic range were clearly evident.

**Figure 1** Effect of the extraction time on the peak area sum of all analytes at 50 ° C with a CAR / PDMS fiber

**Figure 2** Correlation between the extraction and addition of NaCl, as well as the shift of the pH value
